# Endoscopically-assisted transmastoid approach to the geniculate ganglion and labyrinthine facial nerve

**DOI:** 10.1186/s40463-017-0231-1

**Published:** 2017-08-22

**Authors:** Nicholas Jufas, Manohar Bance

**Affiliations:** 1 Division of Otolaryngology – Head and Neck Surgery, Dalhousie University, 3rd Floor Dickson Building, VG Site, QE II Health Sciences Centre, 5820 University Ave, Halifax, NS B3H 2Y9 Canada; 2 Kolling Deafness Research Centre, University of Sydney and Macquarie University, Sydney, Australia; 3 Sydney Endoscopic Ear Surgery (SEES) Research Group, Sydney, Australia

**Keywords:** Surgical Procedure, Endoscopic, Geniculate Ganglion, Decompression, Surgical, Facial Nerve, Semicircular Canals

## Abstract

**Background:**

Endoscopic transcanal approaches to the facial nerve allow excellent exposure of the tympanic facial nerve. This approach becomes limited when access is required to the more proximal geniculate ganglion and labyrinthine portion of the facial nerve. The aim of this report was to determine the feasibility of a transmastoid endoscopically assisted approach to the geniculate ganglion and labyrinthine facial nerve. This is an endoscopic cadaveric dissection and video review at a university anatomical laboratory.

**Methods:**

A total of 12 endoscopic cadaveric dissections were performed. A cortical mastoidectomy and perilabyrinthine air cell removal was performed using an operating microscope. Beyond this, dissection was performed with an endoscope.

**Results:**

In all dissections, an endoscopically assisted transmastoid approach allowed complete access to the geniculate ganglion, and at least 1.5 mm of the distal labyrinthine facial nerve. Further transcrusal drilling through the anterior crus of the superior semicircular canal allowed access to the entire labyrinthine facial nerve.

**Conclusions:**

The entire geniculate ganglion and labyrinthine facial nerve is difficult to access with microscopic techniques. Adding endoscopic visualization allows for complete visualization of the geniculate ganglion. Clinical reports will further strengthen these preliminary cadaveric results.

## Background

The geniculate ganglion and labyrinthine segment of the facial nerve are of importance as they are challenging to access surgically and can be affected by disease. The most common pathologies affecting this region of the facial nerve include inflammation, traumatic injury, cholesteatoma and neoplasms.

This area can be accessed by both the middle fossa and transmastoid approach, each with their own limitations. The transmastoid approach obviates the need for a craniotomy, but is difficult in a poorly pneumatised temporal bone and has been shown able to access the entire labyrinthine segment of the facial nerve in only 60% of patients, mainly through the superior semicircular canal [1].

Although the middle fossa approach offers an alternative, the scarce anatomical landmarks on the floor of the middle cranial fossa can lead to disorientation. Additionally, the inherent craniotomy and temporal lobe retraction to permit an adequate view risks sensorineural and conductive hearing loss, intracranial bleeding, cerebrospinal fluid (CSF) leak and seizures. In addition, in the acute trauma situation, the middle fossa approach may be contra-indicated, if there is concomitant brain injury.

Endoscopic transcanal approaches to the facial nerve provides the most direct approach with minimal bone removal needed allow excellent exposure of the tympanic facial nerve, tympanic portion of the geniculate ganglion and greater superficial petrosal nerve. However the transcanal approach is limited anteriorly by the anterior canal wall and limitation would be expected in visualizing the labyrinthine portion of the geniculate ganglion and the labyrinthine facial nerve. The additional anterior exposure afforded by an extended epitympanotomy may be useful to access these structures. Although an endoscopic transcanal suprageniculate corridor has been recently described, the extent of labyrinthine facial nerve exposure was not quantified [2, 3].

The primary aim of this report was to determine the feasibility of a transmastoid endoscopically assisted approach to the geniculate ganglion and labyrinthine facial nerve. In particular, we wished to evaluate how much of the labyrinthine segment could be visualized without violating the superior semicircular canal. The secondary aim was to analyse the relationship of the geniculate ganglion to the cochleariform process.

## Methods

Approval was obtained under institutional anatomical licencing for cadaveric research for medical and scientific purposes. Six human fresh-frozen cadaveric heads were obtained from an in-house human body donation program. The cadavers had no history of ear surgery, ear disease or trauma.

Using microscopic visualization, a wide post-auricular incision was performed and soft tissues elevated. An extensive cortical mastoidectomy was performed, outlining the otic capsule and exposing the epitympanic portion of the malleus and incus.

Zero, 30 and 45 degree, 3 mm diameter, 14 cm endoscopes, with a SPIES H3-Z three-chip full High Definition (HD) camera, Image1 Connect Processing Module and full HD monitor (Karl Storz Gmbh & Co. KG, Tuttlingen, Germany) were used for visualization. The incudostapedial joint was divided, malleus nipped at the neck and tensor tympani divided. The incus and head of the malleus were removed.

**Fig. 1:**
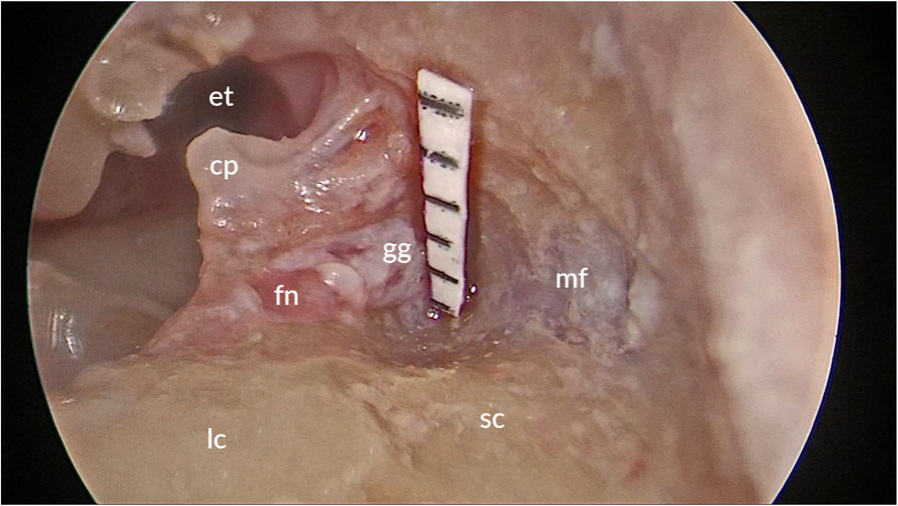
Endoscopic view of specimen (*right ear*), with view of tympanic and labyrinthine facial nerve and geniculate ganglion. Measurement is taken of exposed labyrinthine facial nerve after blue-lining of and prior to entry into the superior semicircular canal. et, Eustachian tube; cp, cochleariform process; fn, facial nerve; gg, geniculate ganglion; lc, lateral semicircular canal; sc, superior semicircular canal; mf, middle cranial fossa dura

Bony removal of the superior aspect of the facial nerve canal above the cochleariform process was then performed using a 2 mm curved diamond burr (Medtronic Inc., Fridley, MN, USA). The epineurium of the facial nerve was exposed proximally along its course to include the entire first genu. Drilling then proceeded proximally along the labyrinthine segment of the facial nerve. Initially the superior semicircular canal was blue-lined but not entered to the maximum extent possible (Fig. 1).

**Fig. 2:**
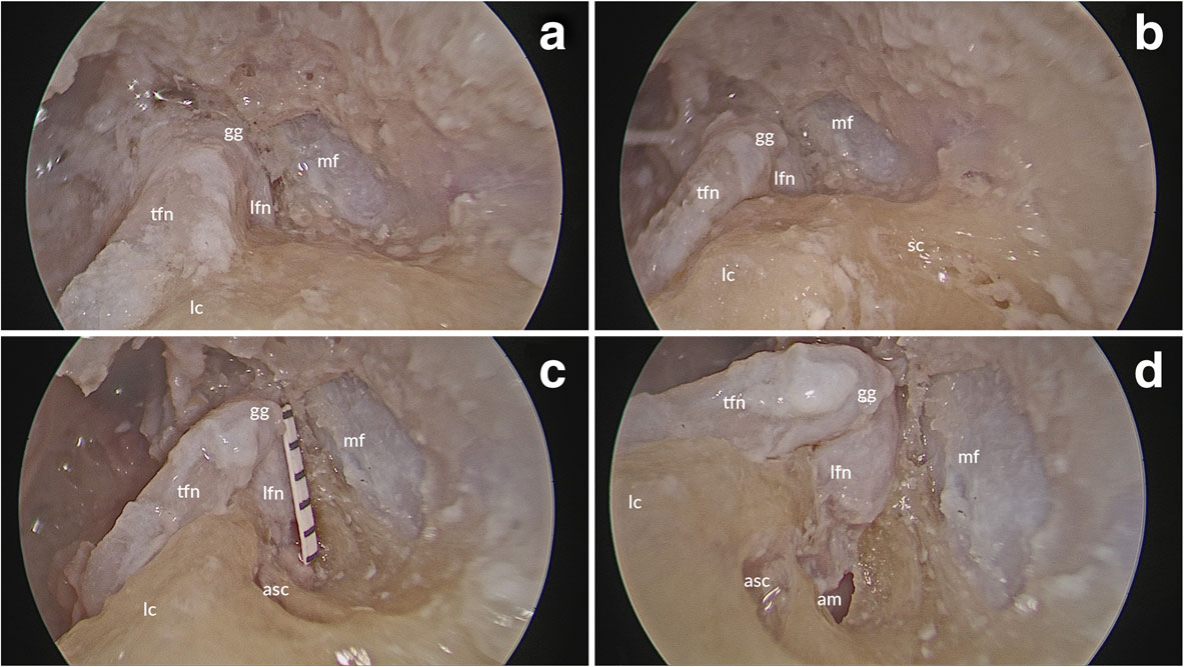
Sequential endoscopic views (**a**-**d**) of specimen (*right ear*), showing progress after displaying air cells, a bony spicule and a protympanic spine in the protympanum. tfn, tympanic facial nerve; gg, geniculate ganglion; lfn, labyrinthine facial nerve; lc, lateral semicircular canal; asc, ampulla of superior semicircular canal; mf, middle cranial fossa dura; am, internal auditory meatus

Further drilling occurred after breaching the anterior crus of the superior semicircular canal in order to attempt to expose the entire labyrinthine portion of the facial nerve via a transcrusal approach. This necessitated, in all cases, a transcrusal approach through the anterior crus of the superior semicircular canal. Exposure along the entire segment was confirmed by noting the dilation of the facial canal as the meatal segment is entered (Fig. 2).

The following aspects of the dissection were noted and measurements taken using a surgical ruler (DeRoyal Industries Inc., Powell, TN, USA) that had been cut into appropriate strips to the nearest 0.25 mm. Two measurements were taken for each, and the results averaged:

- The length of the precochleariform segment, defined as refers to the portion of the tympanic facial nerve lying superiorly and anteriorly to the posterior bony limit of the cochleariform process [3].
- The amount of the geniculate ganglion and length of labyrinthine segment of the facial nerve that could be exposed without breaching the semicircular canals.
- The subsequent length of labyrinthine segment of facial nerve that could be exposed once the superior semicircular canal was breached.

## Results

In all 12 ears dissected, the use of angled endoscopes assisted in increasing the proportion of the geniculate ganglion and labyrinthine segment of the facial nerve that could be seen without breaching the superior semicircular canal.

In all specimens, the geniculate ganglion was able to be fully exposed on its superior aspect, without breaching the superior semicircular canal. The dura of the floor of the middle cranial fossa approached the geniculate ganglion, but in most cases, a thin bony covering was able to be maintained over the dura. Dissection further forward to expose the greater superficial petrosal nerve would have required complete removal of bone and elevation of dura and this was not performed in this study.

In all specimens, the precochleariform segment was measured from the anterior bony limit of the cochleariform process, anteriorly as far forward as possible along the tympanic portion of the facial nerve on its superolateral aspect. The mean precochleariform segment length was 4.52 mm (range 3.75-5.25 mm; standard deviation (SD) 0.47 mm).

Under endoscopic visualisation, and with bluelining the superior semicircular canal to the maximum extent possible, the mean length of the distal labyrinthine segment of facial nerve visible without breaching the superior semicircular canal was 2.39 mm (range 1.88-2.75 mm; SD 0.30 mm).

After exposing the entire labyrinthine segment of the facial nerve to the fundus of the meatal segment via a transcrusal approach and breaching the anterior crus of the superior semicircular canal, the mean length of the entire labyrtinthine segment of facial nerve visible was 4.30 mm (range 3.63-4.75 mm; SD 0.37 mm).

## Discussion

This study has demonstrated that an endoscopically assisted transmastoid approach to the geniculate ganglion and labyrinthine facial nerve is feasible in a cadaver model and defined the limits of accessing the labyrinthine facial nerve with and without transcrusal breach. Exposure of the entire labyrinthine segment requires a transcrusal approach through the superior semicircular canal, whereas just over half of the labyrinthine facial nerve can be exposed without transgression of the labyrinth. This approach may be helpful in treating pathology of this region of the facial nerve, while reducing the risk of complications from more invasive surgery.

Traditional approaches to the geniculate ganglion have either used a transmastoid, middle cranial fossa or a combination of these approaches [4]. Although the middle fossa approach has unique risks and challenges that were discussed earlier, it gives the best chance of hearing preservation as it is the only approach that doesn’t disturb the ossicular chain.

Endoscopes are being increasingly used in otology, with significantly more visualization of every subregion of the middle ear when compared with the microscope [5]. Although endoscopic transcanal approaches afford good exposure of the lateral aspect of the geniculate ganglion and greater superficial petrosal nerve [2], they do not adequately access the more medial aspect of the geniculate ganglion and labyrinthine segment of facial nerve without significant breach of the inner ear, where hearing loss would be expected. In cases where there is significant pre-operative hearing loss, a transcanal transpromontorial approach would certainly afford more direct access.

The labyrinthine facial nerve and geniculate ganglion are often involved in facial nerve pathology, in particular idiopathic inflammatory diseases such as Bell’s palsy [6] and trauma [7], and could be addressed in surgical decompression. The reasoning for this is likely due to the anatomical dimensions of the labyrinthine segment, being the narrowest section of the bony facial nerve canal [8].

It has been demonstrated in this study that it is possible to expose the entire superior aspect of the geniculate ganglion and an average of 2.39 mm of the distal labyrinthine facial nerve, which is over 50% of the entire segmental length, without breaching the labyrinth. We found it necessary to remove the incus and head of malleus, as would be the case in all transcanal and transmastoid approaches. This would need reconstruction with an OCR or a bone anchored hearing aid to bypass the conductive hearing loss.

Although it has been demonstrated to be possible to provide access to the geniculate ganglion without ossicular disruption in a minority of cases, particularly in a very well pneumatised mastoid [4], the experience of this study suggested it would be prudent to pre-emptively disarticulate the ossicles to ensure protection against drill trauma while attempting to expose the labyrinthine facial nerve.

Avoiding the need for a craniotomy inherent in the middle fossa approach is important as this also avoids a number of significant risks and potential complications. Additionally, preservation of the labyrinth by not utilising translabyrinthine or transcochlear approaches implies that there is not necessarily a certain auditory and vestibular loss on the operated side post operatively. Although this approach has been previously demonstrated microscopically [4], our experience using both methods of visualisation in this study was that that the endoscope was invaluable in ensuring that the superior semicircular canal was not inadvertently entered and that the visualised portion of the distal labyrinthine facial nerve was maximised.

This study demonstrated that it is possible to visualise the entire labyrinthine segment of the facial nerve via a transmastoid approach, without a full translabyrinthine drillout. Removing the anterior crus of the superior semicircular canal allowed access to the whole labyrinthine segment. A mean length of 4.30 mm was found across all specimens, which certainly encompasses the well reported length of the segment at 4 mm [8, 9]. Additional confirmation that the meatal segment of the facial nerve had been reached by noting a widening of the bony canal. As previously noted, this necessitated entering and drilling through the superior semicircular canal. A more extensive variation of this has been previously used for access to the petroclival region and petrous apex, where the posterior and superior semicircular canals are carefully plugged at their ampullated ends and common crus, and subsequently drilled through. Obliteration of the canals begins away from the ampullated end and proceeds with a leading edge maintaining a seal with a combination of either bone dust, bone wax and/or fibrin sealant. Preservation of serviceable hearing was possible in at least 58% of patients across published studies [10–12]. It is encouraging that even if complete exposure of the labyrinthine segment is required via a transcrusal approach, it may be possible to preserve sensorineural hearing.

The canal wall is typically taken down in transmastoid approaches to the geniculate ganglion, to aid microscopic visualisation. Benefits of preserving the canal wall, as we have been able to do in our study include a greater ease of placing and securing an ossicular reconstructive prosthesis primarily, greater success in fitting hearing aids in the canal and obviating the need for a meatoplasty. These advantages are minor however and ultimately the decision on whether to take the canal wall down in a transmastoid approach would come down to surgeon experience and preference. If a labyrinthine breach will likely occur either way, then it is arguable whether the more minimal transcrusal approach through the anterior crus of the superior semicircular canal describe herein would provide much advantage. Clinical reports comparing this technique to existing approaches will be needed to clarify this. Nevertheless this paper has been able to define and measure the limits of exposure possible in this novel endoscopically assisted approach.

A final aspect that we wanted to address in this study was the anatomical relationship between the geniculate ganglion and the cochleariform process. There occasionally appears to be a misconception that the geniculate ganglion is superior or immediately anterosuperior to the cochleariform process [13]. This study has shown that while the tympanic segment of the facial nerve is superior, the geniculate ganglion lies on average 4.52 mm, and to a maximum of 5.25 mm more anterior. Although our study had a small sample size of 6 cadavers, this correlates with previous anatomical studies using alternate approaches that have shown a similar result [9, 14].

## Conclusion

The entire geniculate ganglion and labyrinthine facial nerve can be visualised utilising a transmastoid approach with endoscopic assistance. In order to expose the entire labyrinthine facial nerve, a transcrusal approach through the superior semicircular canal is necessary. Exposure of slightly more than the distal half of the labyrinthine segment is possible without transgressing the labyrinth. Clinical reports utilising this technique will verify the preliminary findings and hypotheses of this cadaveric study.

## Abbreviations

CSF: Cerebrospinal fluid
HD: High definition
SD: Standard deviation
